# Human Red Blood Cells Modulate Cytokine Expression in Monocytes/Macrophages Under Anoxic Conditions

**DOI:** 10.3389/fphys.2021.632682

**Published:** 2021-02-18

**Authors:** Antonella Antonelli, Emanuele Salvatore Scarpa, Mauro Magnani

**Affiliations:** Department of Biomolecular Sciences, University of Urbino Carlo Bo, Urbino, Italy

**Keywords:** hypoxia, macrophages, red blood cells, pO_2_ variation, cytokine expression modulation

## Abstract

In the bone marrow (BM) hematopoietic niche, the oxygen tension is usually very low. Such condition affects stem and progenitor cell proliferation and differentiation and, at cellular level regulates hematopoietic growth factors, chemokines and adhesion molecules expression. In turn, these molecules affect the proliferation and maturation of other cellular components of the niche. Due to the complexity of the system we started the *in vitro* investigations of the IL-6, IL-8, TNFα cytokines expression and the vascular endothelial growth factor (VEGF), considered key mediators of the hematopoietic niche, in human macrophages and macrophage cell line. Since in the niche the oxygen availability is mediated by red blood cells (RBCs), we have influenced the anoxic cell cultures by the administration of oxygenated or deoxygenated RBCs (deoxy RBCs). The results reported in this brief paper show that the presence of RBCs up-regulates IL-8 mRNA while IL-6 and VEGF mRNA expression appears down-regulated. This does not occur when deoxy RBCs are used. Moreover, it appears that the administration of RBCs leads to an increase of TNFα expression levels in MonoMac 6 (MM6). Interestingly, the modulation of these factors likely occurs in a hypoxia-inducible factor-1α (HIF-1α) independent manner. Considering the role of oxygen in the hematopoietic niche further studies should explore these preliminary observations in more details.

## Introduction

The bone marrow (BM) is a tissue of complex architecture that is organized into a hematopoietic cell compartment and the stroma, which is mainly composed of fibroblasts, adipocytes, nerves, and the BM’s vascular system (Morrison and Scadden, 2014; Tamma and Ribatti, 2017). In BM thin-walled sinusoidal vessels are highly specialized capillaries with a discontinuous basement membrane and fenestrations that facilitate trafficking of cells and soluble factors between the blood and the BM compartment. This vasculature provides not only a route for mature hematopoietic cells to the peripheral circulation but also a place where hematopoietic progenitors differentiate and set the stage for full reconstitution of hematopoiesis, which maintains in the peripheral blood a constant level of the different blood cell types and components (erythrocytes granulocytes, platelets, lymphocytes, etc.). Oxygen tension (pO_2_) is an important determinant of hematopoietic stem and progenitor cell (HSPC) proliferation and differentiation. Thus, understanding the impact of the BM architectural organization on pO_2_ levels in extravascular hematopoietic tissue is an important biophysical problem. If oxygen concentration in the atmosphere is normally 21%, corresponding to 159 mmHg, in tissues O_2_ levels span from 150 to 20–70 mmHg (2.5–9% oxygen), and markedly lower levels (<1% oxygen) have been described in necrotic tissue sites. The condition of reduced oxygen tension is defined as hypoxia. Reduced oxygen tension (pO_2_ 38 mm Hg, 5% O_2_) has been shown to enhance the production of erythroid, megakaryocytic, and granulocytic-monocytic progenitors *in vitro*. In fact, due to the inaccessibility of bone marrow to direct noninvasive oxygen measurements, some authors have used mathematical modeling of pO_2_ distributions in the bone marrow and speculated that stem cells are located at the region with very low pO_2_ levels (almost anoxic) because this prevents oxygen radicals from damaging these important cells (Chow et al., 2001). Moreover, previous studies have suggested that local oxygen tension determines the location of hematopoietic stem cells (HSCs) in the BM compartment (Parmar et al., 2007; Spencer et al., 2014).

During megakaryopoiesis, megakaryocytes (Mks) differentiate from HSCs and localize in the proximity of the sinusoid blood vessels where they extend long filaments called proplatelets into the blood vessel lumen through the vascular endothelium where platelets, stemming from their terminal ends, are released into the bloodstream by blood shear forces. Despite their critical role in many physiological functions, little is known about the molecular mechanisms involved in platelet production from Mks, or about the pathogenesis of platelet disorders. The characteristics of the environment surrounding Mks play a fundamental role in the regulation of megakaryopoiesis. However, the study of the bone marrow microenvironment *in vivo* has been hampered because of the diffuse three dimensional (3D) nature of its structure and complexity within the bone cavity, especially in humans where invasive approaches are not possible; for this reason, some emerging strategies to obtain information are based on the use of relevant 3D models that offer a scientific approach to mimic and control the physiology of human bone marrow environment within which cells live (Di Buduo et al., 2015; Abbonante et al., 2020). Several authors have contributed in defining niches and mobilization pathways for HSPCs, including the identification of several cell types involved such as osteoblasts, adventitial reticular cells, endothelial cells, monocytic cells, and granulocytic cells and the main factors that anchor HSPCs in the niche and/or induce their quiescence such as vascular cell adhesion molecule (VCAM)-1, CD44, hematopoietic growth factors, e.g., stem cell factor (SCF), chemokines including IL-12 and IL-8 (Richter et al., 2017). A number of cytokines, growth factors, and non-protein metabolites, such as lipids or ions have been demonstrated to play pivotal roles in bone marrow vascular niche regulation (Zhang et al., 2019). Moreover, there is evidence that macrophage cells play an essential role in the maintenance and regulation of BM vascular niche function by secreting large quantities of several molecules with hematopoietic activity, including interleukins that can affect hematopoietic cells. It has been reported that resting macrophages regulate the maturation of Mks and platelet biogenesis releasing mainly IL-8 (D’Atri et al., 2011). It is also known that some sirtuin enzymes, depending by NAD^+^ binding can mediate the transcriptional activation of IL-8 and regulate hypoxia-inducible factor-1 α (HIF-1α) stabilization which is routinely used to screen for hypoxia (Wang et al., 1995; Bauer et al., 2012; Di Girolamo et al., 2013; Edatt et al., 2020). Under normoxic conditions, the α subunit of HIF-1 is hydroxylated by prolyl hydroxylases (PHDs), recognized by the protein product of the von-Hippel-Lindau (VHL) gene, ubiquitinated and degraded by the proteasome (Magnani et al., 1994; Huang et al., 1998; Loboda et al., 2012). In hypoxia the PHDs are not active and consequently HIF-1α is not degraded but can translocate to the nucleus, and can dimerize with the β subunit. The heterodimeric transcription factor induces the transcription of genes mediating cellular adaptation to a low oxygen environment. Macrophages in hypoxic condition also respond with increased IL-6 production that can affect both stromal and hematopoietic cells, for example stimulating Mk growth and maturation *in vitro* as well as increasing Mk ploidy (Murdoch et al., 2005; Tanaka et al., 2014). Expression of also VEGF by macrophages is markedly increased by exposure to hypoxia *in vitro* (Tamura et al., 2020). However, the precise role of macrophages in the regulation of human megakaryo/thrombopoiesis is largely unknown.

In the context of the elucidation of the basic mechanisms of intracellular cross-talks between the different cell components of vascular niche we report preliminary data collected during an *in vitro* study performed with an anoxic cell model about the modulation of the expression of specific cytokines.

## Materials and Methods

### Cell Cultures and Reagents

Human monocyte-derived macrophages were prepared from buffy coats provided by healthy adult volunteers at the Blood Transfusion Center of “*S. Maria* della Misericordia” Hospital in Urbino (PU), Italy. All volunteers signed an informed consent form before donation. Macrophages were prepared by density gradient separation using Lymphoprep solution (specific density, 1.077; Axis-Shield PoC AS, Oslo, Norway). Each experiment was performed with macrophages obtained from a single buffy coat (~50 ml) derived from a single donor. The buffy coat was centrifuged at 150 *g* for 15 min at room temperature to eliminate platelets and serum. The pellet was diluted 1:2 with phosphate buffered saline (PBS) and overlaid on Lymphoprep solution (2:1 ratio) and centrifuged at 300 *g* for 30 min. The ring of peripheral blood mononuclear cells (PBMCs), was isolated, washed twice with PBS and resuspended in RPMI medium supplemented with 10% (v/v) heat-inactivated FBS, 100 U/ml penicillin, 100 μg/ml streptomycin and 2 mM L-glutamine. Essentially, the human macrophages have been obtained from monocytes through their distinguishable ability for plastic adherence. After 24 h in culture in 25 cm^2^ flasks (Cell Star, greiner bio-one), at 37°C in a humidified 5% CO_2_ atmosphere, non-adherent cells were removed by rinsing with cell medium in order to isolate adherent monocytes. The culture media was renewed every 2 days and monocyte-derived macrophages were used at 7th day of adherence. Experiments were performed with 1.5 x 106 macrophages for each condition. MonoMac 6 (MM6) cells, derived from human acute monocytic leukemia (Ziegler-Heitbrock et al., 1988) were obtained from DMSZ GmbH (Braunschweig, Germany) and cultured in 25 cm^2^ flasks at a density of 0.5–1 × 10^6^ cells/ml as described in (Palma et al., 2011). Approximately 2.5 × 10^6^ MM6 cells were used for each experimental condition. MM6, macrophages and RBCs were exposed to anoxia by using an Hypoxia Incubator Chamber (Chamber for generation of a hypoxic environment for tissue culture, StemCell Technologies) flushed with the appropriate 95% N_2_ and 5% CO_2_ gas mixture for 15 min to reach anoxia condition and placed at 37°C in a humidified incubator 95% air, 5% CO_2_. In our experiment design, MM6 cells were treated for 2, 4, 6, 8, 21, and 24 h and human macrophages for 2, 4, 6, and 24 h. Normoxic cells were also maintained in the incubator as control samples. Moreover, in comparison to anoxic condition, some cell samples were also treated with CoCl_2_ (Sigma Aldrich), a widely used chemical hypoxia mimetic model; CoCl_2_ was diluted in complete cell culture media prior to cell stimulation. It has been demonstrated that CoCl_2_ induces hypoxia-regulated genes by stabilizing HIF-1α in normoxia (Yuan et al., 2003).

The human RBCs were prepared from blood collected in heparinized tubes and derived from healthy adult volunteers of Transfusion Center who signed an informed consent. For all the experiments, blood derived from six donors was used. The blood from a single volunteer was used to prepare RBCs for each experiment. RBCs were isolated as already reported (Antonelli et al., 2020) and resuspended in the same buffer at 10% hematocrit before being deoxygenated or not and administered to anoxic MM6 and human macrophages cells for 3 h. After 3 h of incubation with RBCs, the MM6 cells were packed by centrifugation at 900 *g* and RBCs present in the pellet were lysed with sterile distilled water for 3 min. Immediately afterwards, MM6 cells were resuspended in phosphate buffer saline (PBS) and washed twice by centrifugation to eliminate hemoglobin released from RBCs. Instead, RBCs were removed from adherent human macrophages by three washes with PBS buffer. Total cell extracts obtained using specific lysis buffers were processed for real-time quantitative PCR (RT-qPCR) analysis of IL-6, IL-8, TNFα, VEGF, and hypoxia-inducible factor-1α (HIF-1α) mRNA expression or to detect HIF-1α protein by Western blotting analysis.

### Western Blotting Analysis

Cells were lysed in buffer containing Urea 6M, Tiourea 2M, DTT 100 mM, Tris-HCl 30 mM, pH 7.5, Triton 1% and glycerol 9% supplemented with protease inhibitors (cComplete Mini; Roche, Basel, Switzerland); lysates were boiled within 7 min, sonicated twice at 100 Watt for 10 s and cleared by centrifugation at 15,000 × *g* for 10 min, then the supernatants were recovered. The proteins determined by using Bio-Rad Protein Assay (Bio-Rad, Hercules, CA, United States) were resolved by 8% SDS polyacrylamide gel electrophoresis (SDS-PAGE) and afterward transferred onto nitrocellulose membrane (100 V, 70 min at 4°C). The blots were probed with the following primary antibodies: anti-HIF-1α (#14179, monoclonal, recognizing amino acidic residues surrounding Lys460 that is codified by the exon 10 of HIF1A CDS^1^) from Cell Signaling Technology (Danvers, MA, United States); anti-Lamin A/C (#sc-376248, monoclonal) from Santa Cruz Biotechnology (Dallas, TX, United States); anti-β-actin (#VMA00048, monoclonal) from Bio-Rad (Hercules, CA, United States). Immunoreactive bands were detected by horseradish peroxidase (HRP)-conjugated secondary antibodies (Bio-Rad). Peroxidase activity was detected with the enhanced chemiluminescence detection method (WesternBright ECL, Advasta, Menlo Park, CA, United States) using the ChemiDoc MP Imaging System (Bio-Rad). Quantification of the protein bands has been performed using Image Lab analysis software version 5.2.1 (Bio-Rad).

### Real-Time Quantitative PCR

Gene-specific expression analyses were performed as already reported (Scarpa et al., 2020). Fluorescence intensity of each amplified sample was measured with an ABI PRISM 7500 Sequence detection system (Applied Biosystems, Foster City, CA, United States). All measurements were performed at least in triplicate and reported as the average values ± standard deviation of the mean (mean ± SD). Target gene values were normalized with B2M mRNA measurements, and expression data were calculated according to the 2^-ΔΔCT^ method. Primers were designed using Primer 3 Plus, and their sequences are: IL8-F: 5'-TTGCCAAGGAGTGCTAAAGAA-3'; IL8-R: 5'-GCCCTCTTCAAAAACTTCTCC-3'. IL6-F: 5'-AATTCGGTACATCCTCGACGG-3'; IL6-R: 5'-GGTTGTTTTCTGCCAGTGCC-3'. VEGF-F: 5'-TCACAGGTACAGGGATGAGGACAC-3'; VEGF-R: 5'-CAAAGCACAGCAATGTCCTGAAG-3'. B2M-F: 5'- GCCTGCCGTGTGAACCAT-3'; B2M-R: 5'-CATCTTCAAACCTCCATGATGCT-3'. TNFα-F: 5'-GCCCAGGCAGTCA-GATCATCTTC-3'; TNFα-R: 5'-TGCCCCTCAGCTT-GAGGGT-3'. HIF1A-F: 5'-TCTGGGTTGAAACTCAAGCAACTG-3'; and HIF1A-R: 5'-CAACCGGTTTAAGGACACATTCTG-3'.

### Statistical Analysis

The data were expressed as mean ± SD of three independent experiments. Student’s *t*-test and ANOVA test performed with Past Software version 3 were used for statistical analysis of the data; differences between groups were considered statistically significant when *p* < 0.05.

## Results and Discussion

**Figure 1:**
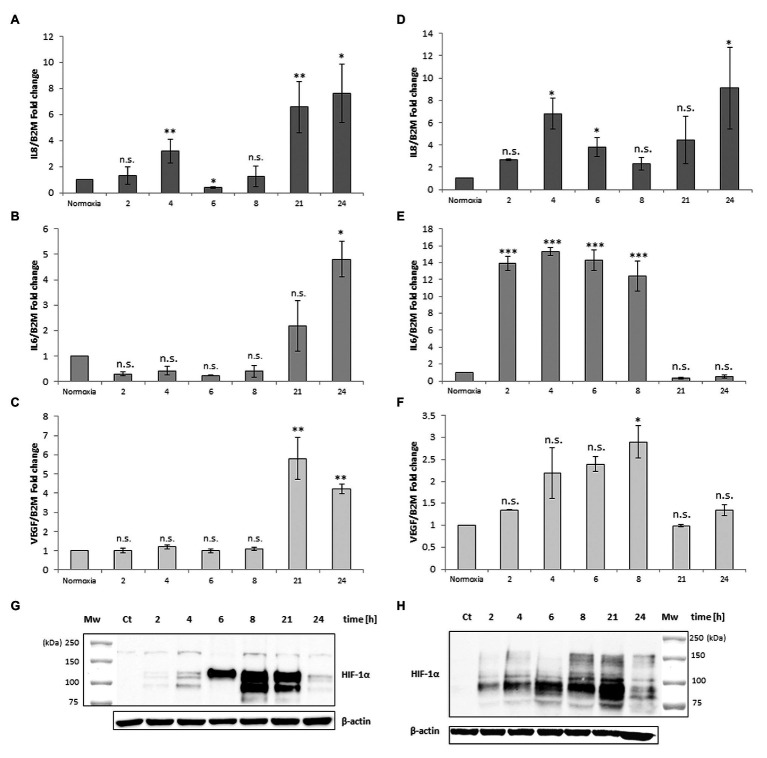
mRNA levels of IL-8, IL-6, and vascular endothelial growth factor (VEGF) in cell extracts of MonoMac 6 (MM6) cells after anoxia (95% N_2_, 5%CO_2_; **A–C**) and 100 μM CoCl_2_ **(D–F)** treatment at different times (hours). Values are expressed as mean ± SD; *n* = 3, ^∗^*p* < 0.05; ^∗∗^*p* < 0.01; ^∗∗∗^*p* < 0.001; n.s., not significant. Western blotting analysis shows the hypoxia-inducible factor-1α (HIF-1α) protein in total extracts after anoxia **(G)** and CoCl_2_ **(H)** treatment of MM6 cells at the indicated times.

Human peripheral blood macrophages and MM6 cells (showing functional features of mature blood monocytes), were considered as cell models to study the effect of anoxia exposure on the expression of some biological factors with a regulatory role in the environment of BM vascular niche. Recent studies show that macrophages respond rapidly to the hypoxia condition by altering their expression of a wide array of genes. Among the various roles played by macrophages, they are also responsible for regulating tissue oxygenation by influencing the formation of new blood vessels and modulating vascular permeability. Notably, in response to hypoxia, macrophages have been shown to induce proangiogenic molecules such as VEGF, and IL-8. Some studies have already shown that HIFs, but not NF-kB, are important transcriptional effectors regulating the responses of macrophages exposed to 18 h hypoxia (Fang et al., 2009). It is currently accepted that resting macrophages, which are relevant components of the BM stroma, release soluble factors that promote megakaryocyte growth, proplatelet production, and platelet release (D’Atri et al., 2011). Here, we investigated the cellular response to anoxia stimulus obtained by using a hypoxic incubator chamber and a gas mixture of 95%N_2_ and 5%CO_2_. Firstly, the results were compared to those obtained with the chemical agent CoCl_2_ which has been shown to mimic the hypoxic conditions in cells by stabilizing the transcription factor HIF-1α. When CoCl_2_ is added to the cell culture, the hydroxylation activity of PHDs is inhibited, therefore HIF-1α protein is not degraded through the ubiquitin/proteasome pathway (Munoz-Sànchez and Chanez-Càrdenas, 2019). Figure 1 shows a representative time-course experiment performed with MM6 cell model exposed to anoxia (Figures 1A–C) and to 100 μM CoCl_2_ (Figures 1D–F). When CoCl_2_ is added to the cell culture, the hydroxylation activity of PHDs is inhibited, therefore HIF-1α protein is not degraded through the ubiquitin/proteasome pathway. mRNA levels of some cytokines such as IL-8, IL-6, and VEGF have been measured. Cells maintained in normoxia (21% O_2_) for the same time period of treatment were used as controls. Under anoxia, an increase of IL-8 mRNA levels (Figure 1A) was found within the 24 h of experimental design; IL-8 expression levels reach values that are about 7-folds higher than controls; the highest expression values are at 4 and 24 h of incubation (4 h: 3.21 ± 0.93, *p* = 0.004; 24 h: 7.62 ± 2.24, *p* = 0.015) similarly to what happens with the CoCl_2_ treatment (Figure 1D) performed in the same time range (4 h: 6.81 ± 1.41, *p* = 0.025; 24 h: 9.10 ± 3.64, *p* = 0.018). Moreover, IL-6 expression levels significantly increase after 24 h of anoxia exposure (4.81 ± 0.70, *p* = 0.029; Figure 1B), whereas in CoCl_2_-treated cells the IL-6 levels increase already from 2 h with a peak of expression at 4 h (15.32 ± 0.44, *p* < 0.001), Figure 1E. Furthermore, VEGF expression levels remarkably increase only after 21 h and 24 h anoxia treatment (Figure 1C), with a peak at 21 h (5.80 ± 1.10, *p* = 0.003) and also after CoCl_2_ treatment (Figure 1F), with a maximum peak at 8 h (2.90 ± 0.37, *p* = 0.046). At the same time, we have evaluated by western blot analyses the content of HIF-1α protein in cell lysates of MM6 cells exposed to anoxia (Figure 1G) and CoCl_2_ (Figure 1H). It is evident that HIF-1α expression was remarkably induced under anoxic condition after 6 h of exposure with an increase of the protein levels up to 21 h followed by a return toward basal levels at 24 h (Figure 1G, Table 1). Similar results obtained after CoCl2 treatment are shown in Figure 1H where the HIF-1α protein appears in different isoforms (in the range of 75–100 kDa), as already described in literature (Monsef et al., 2010), Figure 1H and Table 1. In fact, it is not uncommon to detect protein bands other than at the one expected at 120 kDa; different forms of HIF-1α protein detectable after CoCl_2_ treatment, have been already described (Rana et al., 2019).

**Table 1:** Quantification of the HIF-1α protein bands in the immunoblots reported in Figures 1–3.

Figure 1G	Net Intensity (A.U.)	SD	*p* value	Figure 1H	Net intensity (A.U.)	SD	*p* value	Figure 3I	Net intensity (A.U.)	SD	*p* value
Control	0.0016	0.0004		Control	0.0006	0.0001		Control	0.0246	0.0071	
Anoxia 2 h	0.0778	0.0114	0.294	CoCl_2_ 2 h	0.0307	0.0073	0.002^**^	Anoxia 21 h	0.2047	0.0570	0.025^*^
Anoxia 4 h	0.3220	0.1207	0.002^**^	CoCl_2_ 4 h	0.0810	0.0250	0.005^**^	Anoxia + RBCs	0.0544	0.0236	0.090
Anoxia 6 h	3.7122	1.4873	0.006^**^	CoCl_2_ 6 h	0.0897	0.0250	0.003^**^	Anoxia + RBCs deox	0.1095	0.0349	0.034^*^
Anoxia 8 h	6.0074	2.7509	0.008^**^	CoCl_2_ 8 h	0.1323	0.0443	0.007^**^	Anoxia + Air	0.0231	0.0121	0.648
Anoxia 21 h	3.1870	1.4013	0.006^**^	CoCl_2_ 21 h	0.2883	0.1287	0.003^**^	CoCl_2_	0.4851	0.1962	0.027^*^
Anoxia 24 h	0.1643	0.0543	0.181	CoCl_2_ 24 h	0.0303	0.0128	0.016^*^	Figure 3J	Net intensity (A.U.)	SD	*p* value
Figure 2G	Net intensity (A.U.)	*SD*	*p* value	Figure 2H	Net intensity (A.U.)	SD	*p* value	Control	0.0011	0.0004	
Control	0.0065	0.0022		Control	0.1348	0.0356		Anoxia + Air	0.0017	0.0011	0.297
Anoxia 2 h	0.0173	0.0070	0.004^**^	CoCl_2_ 2 h	0.9576	0.1652	<0.001^***^	Anoxia 6 h	0.1505	0.0374	0.020^*^
Anoxia 4 h	0.0458	0.0255	0.024^*^	CoCl_2_ 4 h	1.0009	0.0931	<0.001^***^	Anoxia + RBCs	0.0062	0.0006	0.061
Anoxia 6 h	0.0150	0.0078	0.146	CoCl_2_ 6 h	0.4786	0.1036	0.005^**^	Anoxia + RBCs deox	0.0398	0.0169	0.026^*^
Anoxia 24 h	0.0199	0.0084	0.004^**^	CoCl_2_ 24 h	0.2218	0.0652	0.112	CoCl_2_	0.8187	0.2710	0.035^*^

**Figure 2:**
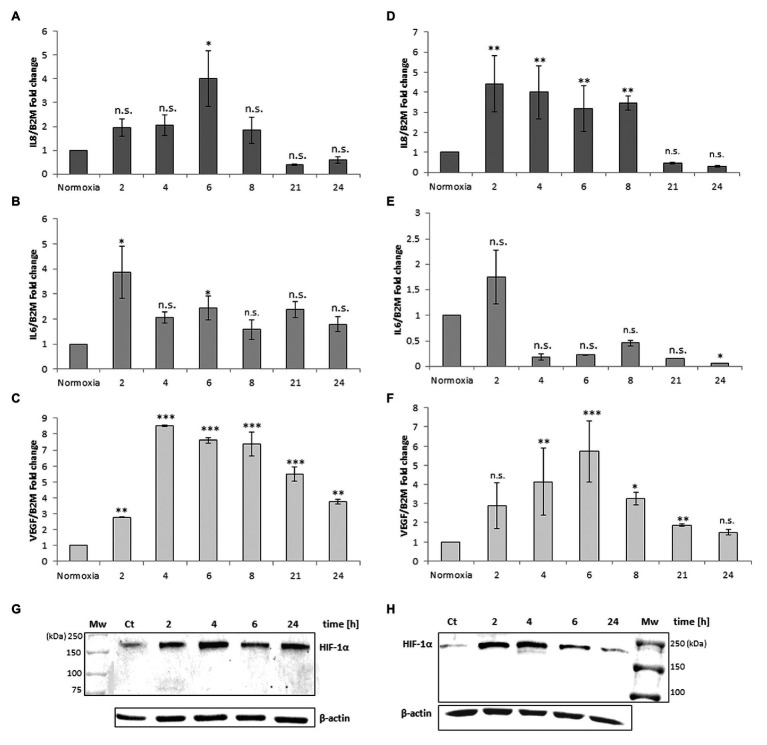
mRNA levels of IL-8, IL-6 and VEGF in cell extracts of human macrophages from peripheral blood after anoxia (95% N_2_, 5%CO_2_; **A–C**) and 100 μM CoCl_2_ **(D–F)** treatment at different times (hours). Values are expressed as mean ± SD; *n* = 3, ^∗^*p* < 0.05; ^∗∗^*p* < 0.01; ^∗∗∗^*p* < 0.001; n.s. not significant. Western blotting analysis shows the HIF-1α protein in total extracts after anoxia **(G)** and CoCl_2_ **(H)** treatment of human macrophages at the indicated times.

**Figure 3:**
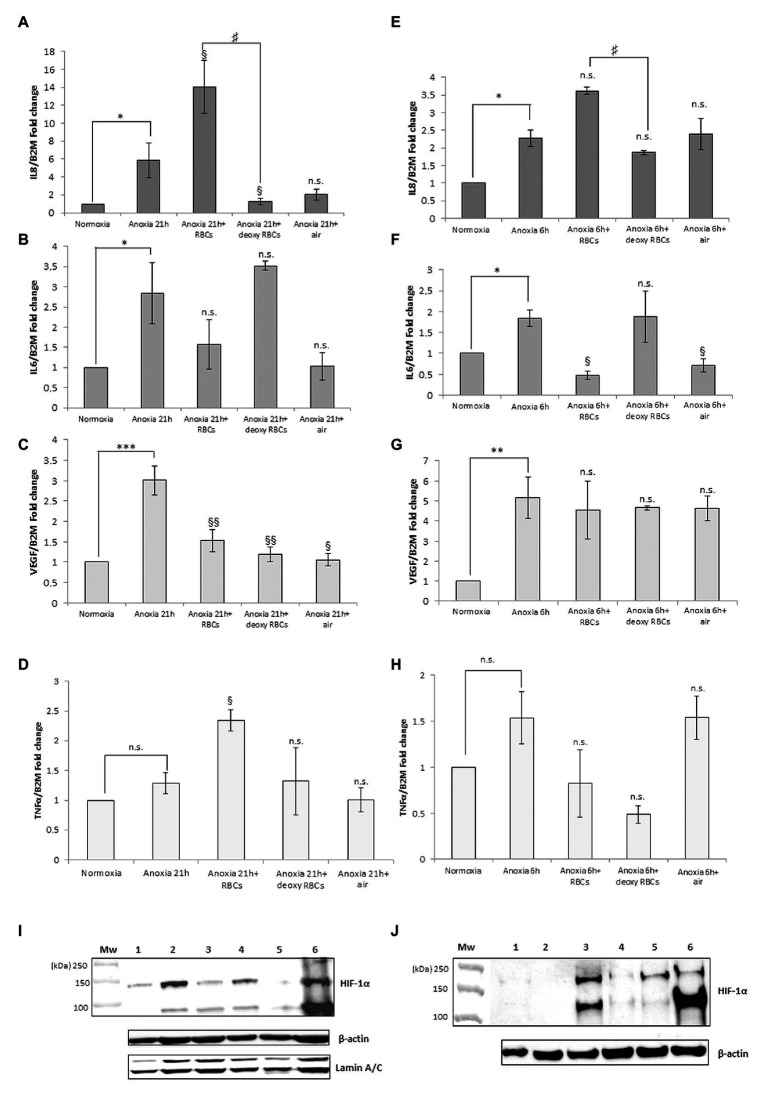
mRNA levels of IL-8, IL-6, VEGF and TNFα in cell extracts of MM6 cells **(A–D)** and human macrophages **(E–H)** after the incubation with red blood cells (RBCs; 10% Ht) deoxygenated or not. Values are expressed as mean ± SD; *n* = 3; ^∗^*p* < 0.05, ^∗∗^*p* < 0.01, ^∗∗∗^*p* < 0.001 when compared with normoxic samples. ^§^*p* < 0.05, ^§§^*p* < 0.01 when compared with anoxic samples. ^#^*p* < 0.05 when anoxia + RBCs samples were compared with anoxia + deoxy RBCs samples. Western blotting analysis shows the HIF-1α protein in total extracts of MM6 cells (**I**, 1. Control 2. Anoxia 21 h 3. Anoxia 21 h + RBCs 3 h 4. Anoxia 21 h + deoxy RBCs 3 h 5. Anoxia 21 h + air 3 h; and 6. 100 μM CoCl_2_ 21 h) or human macrophages (**J**, 1. Control 2. Anoxia 6 h + air 3 h 3. Anoxia 6 h 4. Anoxia 6 h + RBCs 3 h 5. Anoxia 6 h + deoxy RBCs 3 h; and 6. 100 μM CoCl_2_ 6 h) after incubation with RBCs (10% Ht) deoxygenated or not.

The quantification of HIF-1 protein was performed by densitometry measurements using Image Lab software version 5.2.1 (Bio-Rad). Figures 1G,H, 3I refer to MM6 cells and Figures 2G,H, 3J to human macrophages. Statistical analyses were performed with Past 3 Software, using ANOVA method for data of Figures 1G,H, 2G,H and paired student’s *T*-test for Figures 3I,J. A.U., arbitrary units.

* *p* < 0.05.

** *p* < 0.01.

*** *p* < 0.001.

The same experimental design was performed with monocyte-derived macrophages isolated from human peripheral blood. Figure 2 shows a different response to the anoxia or CoCl_2_ stimuli with respect to leukemia MM6 cells. After 6 h of anoxia exposure the IL-8 mRNA level increases of about 4-fold (4.02 ± 1.16, *p* = 0.047) respect to normoxia value (Figure 2A) whereas with CoCl_2_ treatment the maximum expression occurs at shorter times (2 h; 4.43 ± 1.40, *p* = 0.003; Figure 2D); in both cases the mRNA expression decreases within the 21 h. IL-6 mRNA expression seems to augment after already 2 h of CoCl_2_ (Figure 2E) or anoxia (Figure 2B) treatment reaching values of about 1.5-fold (1.76 ± 0.53, *p* = 0.603) and 4-fold (3.86 ± 1.05, *p* = 0.041) increase, respectively. VEGF expression appears to have a similar trend when macrophages are exposed to both stimuli (Figures 2C,F) which induce a remarkable increase of mRNA levels in the range of 4–6 h of treatment (peak at 4 h for anoxia; 8.49 ± 0.05, *p* < 0.001; peak at 6 h for CoCl_2_, 5.74 ± 1.59, *p* < 0.001).

The results obtained from these analyses are in accordance with literature; for example, it is well known that human macrophages respond to oxygen stress such as an anoxia exposure with augmented production of IL-8 (Metinko et al., 1992; Rydberg et al., 2003).

Western blotting analysis of HIF-1α protein in the macrophages extracts obtained after anoxia (Figure 2G) and CoCl_2_ (Figure 2H) stimuli at the indicated times showed increased amounts of the protein, as 200 kDa band, already from the 2 h of treatment (Table 1). HIF-1α protein can be still detected at 24 h after anoxia exposure while it decreases at the same time after CoCl_2_ treatment. After the evaluation of cell response to anoxia stimulus establishing the highest peaks of gene expression of these specific cytokines (6 h for human macrophages and 21 h for MM6 cells), we have *in vitro* studied the impact of RBCs on these cell lines in order to understand if a variation of pO_2_ level in anoxic environment could alter or regulate the expression of these soluble factors. In fact, oxygen transport to the hematopoietic niche is mediated by RBCs and RBC oxygenation represents more closely the niche oxygen availability rather than the simple oxygen concentration in the tissue cultures.

Figure 3 shows the IL-8, IL-6, VEGF and TNFα mRNA levels obtained after the addition of RBCs at 10% hematocrit for 3 h to MM6 (Figures 3A–D) and human macrophage cells (Figures 3E–H), respectively. The data were compared with results obtained after the administration of deoxy RBCs to MM6 cells or human macrophages incubated at the same condition of cells treated with native RBCs. It is evident that a further increase in IL-8 expression levels in anoxic MM6 cells incubated with native RBCs occurs (anoxia 21 h + RBCs, 14.08 ± 2.93 vs. anoxia 21 h; 5.88 ± 1.92, *p* = 0.048, Figure 3A), but this treatment did not lead to an increase in IL-6 expression levels, rather to a decrease (anoxia 21 h + RBCs 1.565 ± 0.62 vs. anoxia 21 h, 2.84 ± 0.75, *p* = 0.48, Figure 3B). Moreover, VEGF mRNA levels significantly decrease (anoxia 21 h + RBCs 1.525 ± 0.28 vs. anoxia 21 h 3.01 ± 0.35, *p* = 0.004, Figure 3C). In addition, the incubation of MM6 cells with deoxy RBCs did not lead to an increase of IL-6 and VEGF expression levels (for IL-6; anoxia 21 h + deoxy RBCs 3.52 ± 0.11 vs. anoxia 21 h, 2.84 ± 0.75, *p* = 0.56). Concerning VEGF mRNA levels, the values decreased when deoxy RBCs were administered to anoxic MM6 (anoxia 21 h + deoxy RBC 1.19 ± 0.18 vs. anoxia 21 h, 3.01 ± 0.35, *p* = 0.007) as also after reoxygenation of anoxic cells (anoxia 21 h + air, 1.05 ± 0.15 vs. anoxia 21 h, 3.01 ± 0.35, *p* = 0.039, Figure 3C). In addition, IL-8 mRNA levels appear also decreased after the treatment with deoxy RBCs compared to values obtained with anoxia exposure (anoxia 21 h + deoxy RBCs 1.23 ± 0.36 vs. anoxia 21 h 5.88 ± 1.92, *p* = 0.037). Moreover, the value of IL-8 mRNA level of anoxic MM6 cells treated with deoxy RBCs was lower than value of cells treated with native RBCs (anoxia 21 h + RBCs 14.08 ± 2.93, *p* = 0.047), Figure 3A. Interestingly, Figure 3I that relates to western blot analysis of the same cell samples, shows a strong decrease of HIF-1α band protein when anoxic MM6 cells are treated with native RBCs (lane 3), similarly to reoxygenated MM6 cells (lane 5), while the protein band is evident after 21 h anoxia exposure (lane 2) or after 100 μM CoCl_2_ treatment (lane 6), as expected. The administration of deoxygenated RBCs to anoxic MM6 cells does not seem to lead to the same result, since HIF-1α protein band is maintained (lane 4). Densitometric analysis of Figure 3I has been reported in Table 1. In Figure 3I the blot of lamin protein as control protein was reported in addition to actin, in order to show that the protein content derived only from MM6 cells and not from RBCs. Figure 3 shows in similar way the levels of IL-8, IL-6, TNFα and VEGF mRNA expression in human macrophages as response to the same stimuli; IL-8 mRNA level appears to increase in anoxic cells treated with native RBCs (anoxia 6 h + RBCs, 3.62 ± 0.11-folds vs. anoxia 6 h, 2.27 ± 0.24, *p* = 0.61, Figure 3E) whereas IL-6 levels significantly decrease (anoxia 6 h + RBCs 0.47 ± 0.10-folds vs. 1.84 ± 0.20, *p* = 0.045; Figure 3F). Moreover, VEGF mRNA levels do not seem to change (4.54 ± 1.43 vs. 5.16 ± 1.04, *p* = 0.56), Figure 3G. On the contrary, the treatment with deoxy RBCs results in the expression levels of the target genes which do not differ from those found in the cells exposed to only anoxia. In fact, no significant increase of neither IL-8 (Figure 3E) nor VEGF (Figure 3G) mRNA values were found respect to those found in the anoxic cells (for IL-8; anoxia 6 h + deoxy RBC, 1.87 ± 0.06 vs. anoxia 6 h 2.27 ± 0.24, *p* = 0.29; for VEGF; 4.65 ± 0.10 vs. anoxia 6 h 5.16 ± 1.04, *p* = 0.26). Moreover, IL-6 mRNA expression after anoxia exposure (Figure 3F) significantly decreases after the re-oxygenation of cells, and it does not change after incubation with deoxy RBCs (anoxia 6 h + air 0.71 ± 0.16 and anoxia 6 h + deoxy RBCs 1.87 ± 0.62 vs. anoxia 6 h 1.84 ± 0.20, with *p* = 0.013 and *p* = 0.88, respectively). In addition, the increase of IL-8 mRNA value (Figure 3E) after administration of native RBCs is significantly different respect to value obtained with deoxy RBCs (anoxia 6 h + RBCs 3.62 ± 0.11 vs. anoxia 6 h + RBC deoxy 1.87 ± 0.06, *p* = 0.042). Furthermore, it appears that anoxia treatment leads to slight increase of TNFα mRNA levels in both MM6 (1.29 ± 0.18-fold) and human macrophages (1.54 ± 0.28-fold) respectively, Figures 3D,H. Moreover, the administration of native RBCs seems to further significantly increase these values (2.34 ± 0.17-fold, *p* = 0.042) in anoxic MM6 cells, while the ddeoxy RBCs do not (1.32 ± 0.57-fold, *p* = 0.756), Figure 3D. Instead, the treatments with both native and deoxy RBCs to anoxic human macrophages leads to a not significant decrease of TNFα expression levels (0.82 ± 0.36-fold and 0.48 ± 0.09-fold, respectively), Figure 3H. Although increased levels of TNFα mRNA after RBCs treatment were evidenced, they did not reach values obtained for IL-8 cytokine; however, the data could be interesting considering that it is known that TNFα is the principal cytokine driving the adhesion of MM6 cells to endhotelial cells as already reported (Schuettpelz and Link, 2013; Poussin et al., 2014). The Supplementary Tables 1, 2 report the RTqPCR data of Figure 3.

Figure 3J, showing analysis of HIF-1α protein in human macrophage samples, evidences a response to these treatments similar to MM6 cells. In fact, the incubation of macrophages exposed 6 h to anoxia with native RBCs leads to the disappearance of HIF-1α in total cell extracts (lane 4) and this partially occurs with the deoxy RBCs (lane 5). Densitometric analysis of Figure 3J has been reported in Table 1. The presence of different HIF-1α protein isoforms revealed by western blotting analysis (Figures 1G,H; Figures 2G,H; Figures 3I,J) could be explained by their possible post-translational modifications. It is not unusual to find in literature several articles concerning the presence of a broad range of HIF-1α isoforms, commonly reported in both healthy and tumor cells (Monsef et al., 2010). Several studies report on HIF-1α isoforms lacking several exons than the wild type full length isoform. Some of these isoforms encode cytoplasmic HIF-1α protein or proteins with altered transcriptional activity compared to the wild type protein (Gothié et al., 2000; Chun et al., 2001, 2002; Depping et al., 2004; Lukashev and Sitkovsky, 2008). Different HIF-1α isoforms, such as HIF-1α 1.2 which is 59 amino acids shorter (86 KDa) than wild type HIF-1α (93 KDa; Depping et al., 2004), HIF1α 1.3 which encodes a functional protein of 95 KDa (Lukashev and Sitkovsky, 2008), and isoforms lacking either exon 12 (62 KDa) or exon 14 (82 KDa; Gothié et al., 2000; Chun et al., 2001) or exons 11 and 12 (58 KDa; Chun et al., 2002) were found. Importantly, the anti-HIF-1α antibody used in our analyses can recognize also the HIF-1α protein isoforms codified by the alternative splicing variants that act as dominant negative HIF-1α isoforms (Chun et al., 2001, 2002), which are able to inhibit the activity of wild type full length HIF-1α. The different molecular weights showed by our immunoblots could be explained by both alternative splicing events or by different post-translational modifications including phosphorylation, S-nitrosylation and acetylation (Toffoli et al., 2007; Geng et al., 2012; Sanhueza et al., 2020). Moreover, the dimeric protein that appears at a position of approximately 200 kDa could be explained by the presence of HIF-1 α complexed with other proteins or factors (e.g., the constitutively expressed HIF-1β-subunit, or enzymes such as VHL).

Our data indicate that RBCs could up-regulate IL-8 mRNA and down-regulate IL-6 mRNA and VEGF mRNA expression in a way that is independent of HIF-1α in human macrophages in anoxic condition; similarly, this occurs also for the human monocytic MM6 cells that respond also with an increased TNFα mRNA expression.

Moreover, we investigated if the expression of HIF-1 α mRNA levels could be changed in anoxic MM6 and macrophage cells treated with RBCs to provide another way supporting the not involvement of HIF-1α in the up-regulation of IL-8. These results are reported in Supplementary Figure 1, showing a decrease of HIF-1α mRNA levels in cell extracts of human MM6 and macrophage anoxic cells treated with native and deoxygenated RBCs; specifically, for MM6 cells 0.57 ± 0.29-fold (*p* = 0.157, Supplementary Figure 1A) and for macrophages 0.75 ± 0.14-fold (*p* = 0.115, Supplementary Figure 1B) when native human RBCs were administered. When deoxygenated RBCs were administered to MM6 cells, 0.79 ± 0.12-fold (*p* = 0.102, Supplementary Figure 1A) and to human macrophages 0.84 ± 0.21-fold (*p* = 0.295, Supplementary Figure 1B) were found. Thus, HIF-1α mRNA levels were not significantly different when compared with levels of anoxic cells (MM6 cells 0.62 ± 0.21-fold, Supplementary Figure 1A; human macrophages 0.78 ± 0.05-fold, Supplementary Figure 1B). Since both HIF-1α mRNA and corresponding protein levels decrease in cells treated with native RBCs, we can hypothesize that HIF-1α transcription factor could not be directly involved in the increase of IL-8 expression levels. In literature, some works reported evidences that link the upregulation of IL-8 with the increased recruitment of some transcriptional factors binding IL-8 promoter, like Egr-1 (Singha et al., 2014). Probably, the different expression peaks of IL-8 mRNA in MM6 (21 h) and macrophage cells (6 h) could be explained by the fact that Egr-1 or other transcription factors accumulate early in macrophages after exposure to hypoxic/anoxic conditions and lately in monocytic leukemia cells (Elbarghati et al., 2008).

We think that RBCs, as modulator of pO_2_, could interact with these cell systems through mechanisms that would be interesting to study.

Since our data indicate that native RBCs administrated to monocyte-macrophage cell lines induce a specific modulation of IL-8 mRNA expression, it could be useful to understand which are the transcription factors on which these specific cellular responses depend. This aspect is important considering the putative role that this interleukin plays in vascular niche of BM. It was shown that, as other soluble factors, the IL-8 has a pleiotropic role and affects, directly or indirectly, different cellular pathways such as hematopoietic differentiation, cell survival and angiogenesis (Dudek et al., 2003; Aronovich et al., 2013; Poulos et al., 2013). On the other hand, it is known that the maturation of RBCs involves some interactions with macrophages, first during their development in the BM, later in the blood stream with macrophages found in the liver and spleen. These interactions are essential to maintain RBC homeostasis or to ensure the correct removal of aged or damaged RBCs. Our studies could aid to reveal how some biological factors, derived from macrophage and RBC interactions, play a role in BM vascular niche. Considering that contacts take place between macrophage and erythroblastic islands and that erythrocytic cells are capable of migrating toward BM sinusoids as erythroid precursors, it is also reasonable to think that the RBC-macrophages interactions can affect or regulate the function of the other cell components in bone vascular niche, such as megakaryocytic cells associated with the BM vasculature (Machlus and Italiano, 2013). Several researchers are attempting to clarify these aspects studying the dynamic interactions between cellular and molecular components of the BM vascular niche (Zhang et al., 2019); co-culture cell models, where different cells types (for example erythrocytes, macrophages, megakaryocytes and endothelial cells) coexist together, represent versatile tools for investigating these cellular interactions. For example, the biocompatible bioengineered tissue-models, such as 3D BM device mimicking the different features of the BM environment (Di Buduo et al., 2015; Abbonante et al., 2020) are promising approaches to study megakaryocyte function; once in contact with the biomaterial, megakaryocytes extend proplatelets into the perfused culture medium, mimicking blood shear stress. Therefore, the possibility to take advantage of this perfusion bioreactor chamber mimicking *ex vivo* the vascular niche, will allow to transfer in a 3D cell model the results herein reported in order to better understand how the RBCs can influence the intercellular cross-talk in the vascular niche.

## Conclusion

Herein, we investigated the modulation of mRNA expression of few key cytokines (IL-6, IL-8, VEGF, and TNFα) and the possible role of HIF-1α transcription factor, in the early response to the stimuli considered in our experimental cell model; the data indicated the not involvement of HIF-1α in the regulation of these specific cytokines. HIF-1 independent cellular pathways have been reported in solid tumor such as the glioblastoma (Tardòn et al., 2020). Other mechanisms that do not involve HIF-1α have already been described, thus elucidating a novel HIF-independent point of control of cellular metabolism, energetics, and post-transcriptional gene regulations by O_2_, such as mTOR inactivation, or the activation of NF-kB through reactive oxygen species (ROS; Arsham et al., 2003; Mizukami et al., 2005, 2007; Lluis et al., 2007; Park et al., 2012). We can speculate that the treatment of anoxic cells with RBCs leads to a remarkable decrease of HIF-1α, which acts as a repressor of IL-8 (Loboda et al., 2012). This point would explain the observed increase of IL-8 mRNA levels. Furthermore, it was evidenced that, in a model of human glioblastoma, the hypoxia-induced accumulation of HIF-1α was correlated with an increase of IL-6 levels (Xue et al., 2016). Therefore, in our cell model the RBC-mediated decrease of HIF-1α could explain the decrease of IL-6 mRNA levels. Other factors such as HIF-2α, HIF-3α, AP-1, C/EBP and NF-kB may play a role in hypoxia (Shih and Claffey, 1998; Hirani et al., 2001; Rius et al., 2008; Palazon et al., 2014), while ATF-4 and Egr-1 are hypoxia responsive factors in macrophages, but only after early exposures (Elbarghati et al., 2008). In conclusion, a number of transcription factors work together in a tightly regulated fashion to control macrophage activities in hypoxic condition. Further studies are necessary to examine the role of those players under the experimental conditions investigated.

## Data Availability Statement

The authors acknowledge that the data presented in this study must be deposited and made publicly available in an acceptable repository, prior to publication. Frontiers cannot accept a manuscript that does not adhere to our open data policies.

## Ethics Statement

The authors state that they have obtained appropriate institutional review board approval or have followed the principles outlined in the Declaration of Helsinki for all human experimental investigations. In addition, for investigations involving the use of human blood, an informed consent has been obtained from the subjects involved, on the basis of official document of accordance with the Transfusion Center of “*S. Maria* della Misericordia” Hospital in Urbino (PU), Italy.

## Author Contributions

AA and MM defined the study and planned the experiments. AA and ES performed experiments and data acquisition and analysis. AA wrote the manuscript. MM revised the manuscript. All authors contributed to the article and approved the submitted version.

### Conflict of Interest

The authors declare that the research was conducted in the absence of any commercial or financial relationships that could be construed as a potential conflict of interest.

## Footnotes

^1^https://www.ncbi.nlm.nih.gov/nuccore/NM_001530.4
