# Supplementary material for: Human Red Blood Cells Modulate Cytokine Expression in Monocytes/Macrophages Under Anoxic Conditions
Source: Front Physiol. 2021 Feb 18;12:632682. doi: 10.3389/fphys.2021.632682 (PMC7930825; doi:10.3389/fphys.2021.632682)
Supplement: Supplementary file 1 [file Table_1.pdf]

Supplementary TABLE 1 Summary of statistical analysis of IL-8, IL-6, VEGF and TNFα mRNA levels expressed as (fold) changes relative to experiments performed with MonoMac6 cells at 21 hours (time corresponding to the maximum MM6 cells response to anoxia stimulus) and showed in **Figure 3** (panels A, B, C, D).

MonoMac6 cells					
**IL-8**	**Fold**	**SD**	***p***	***p***	***p***
Control	1	0	*vs Control*		
Anoxia 21h	5.88	1.92	0.038	*vs Anoxia 21h*	
Anoxia 21h+RBCs	14.08	2.93	<0.001	0.048	*vs Anoxia 21h+RBCs*
Anoxia 21h+ deoxy RBCs	1.23	0.36	0.35	0.037	0.047
Anoxia 21h+air	2.04	0.63	0.95	0.07	0.028
**IL-6**					
Control	1	0	*vs Control*		
Anoxia 21h	2.84	0.75	0.011	*vs Anoxia 21h*	
Anoxia 21h+RBCs	1.57	0.62	0.44	0.48	*vs Anoxia 21h+RBCs*
Anoxia 21h+ deoxy RBCs	3.52	0.11	0.04	0.56	0.57
Anoxia 21h+air	1.03	0.34	0.67	0.71	0.94
**VEGF**					
Control	1	0	*vs Control*		
Anoxia 21h	3.01	0.35	0.005	*vs Anoxia 21h*	
Anoxia 21h+RBCs	1.53	0.28	0.03	0.004	*vs Anoxia 21h+RBCs*
Anoxia 21h+ deoxy RBCs	1.19	0.18	0.10	0.007	0.21
Anoxia 21h+air	1.05	0.15	0.51	0.04	0.39
**TNFα**					
Control	1	0	*vs Control*		
Anoxia 21h	1.29	0.18	0.81	*vs Anoxia 21h*	
Anoxia 21h+RBCs	2.34	0.18	0.044	0.042	*vs Anoxia 21h+RBCs*
Anoxia 21h+ deoxy RBCs	1.32	0.57	0.76	0.65	0.22
Anoxia 21h+air	1.01	0.21	0.43	0.57	0.14

Supplementary TABLE 2 Summary of statistical analysis of IL-8, IL-6, VEGF and TNFα mRNA levels expressed as (fold) changes relative to experiments performed with human macrophages at 6 hours (time corresponding to the maximum human macrophage response to anoxia stimulus) and showed in **Figure 3** (panels E, F, G, H).

**Human macrophages**					
**IL-8**	**Fold**	**SD**	***p***	***p***	***p***
Control	1	0	*vs Control*		
Anoxia 6h	2.27	0.24	0.047	*vs Anoxia 6h*	
Anoxia 6h+RBCs	3.62	0.11	0.026	0.61	*vs Anoxia 6h+RBCs*
Anoxia 6h+ deoxy RBCs	1.87	0.06	0.097	0.29	0.042
Anoxia 6h+air	2.39	0.45	0.133	0.88	0.50
**IL-6**					
Control	1	0	*vs Control*		
Anoxia 6h	1.84	0.20	0.043	vs *Anoxia 6h*	
Anoxia 6h+RBCs	0.47	0.10	0.46	0.045	*vs Anoxia 6h+RBCs*
Anoxia 6h+ deoxy RBCs	1.88	0.62	0.41	0.88	0.89
Anoxia 6h+air	0.71	0.16	0.42	0.013	0.26
**VEGF**					
Control	1	0	*vs Control*		
Anoxia 6h	5.16	1.04	0.007	*vs Anoxia 6h*	
Anoxia 6h+RBCs	4.54	1.43	0.016	0.56	*vs Anoxia 6h+RBCs*
Anoxia 6h+ deoxy RBCs	4.65	0.10	p<0.001	0.26	0.69
Anoxia 6h+air	4.63	0.63	0.0011	0.30	0.89
**TNFα**					
Control	1	0	*vs Control*		
Anoxia 6h	1.54	0.28	0.056	*vs Anoxia 6h*	
Anoxia 6h+RBCs	0.82	0.36	0.397	0.155	*vs Anoxia 6h+RBCs*
Anoxia 6h + deoxy RBCs	0.49	0.09	0.903	0.549	0.727
Anoxia 6h+air	1.54	0.24	0.051	0.979	0.159
