# Supplementary material for: Human Red Blood Cells Modulate Cytokine Expression in Monocytes/Macrophages Under Anoxic Conditions
Source: Front Physiol. 2021 Feb 18;12:632682. doi: 10.3389/fphys.2021.632682 (PMC7930825; doi:10.3389/fphys.2021.632682)
Supplement: Supplementary file 2 [file Data_Sheet_1.pdf]

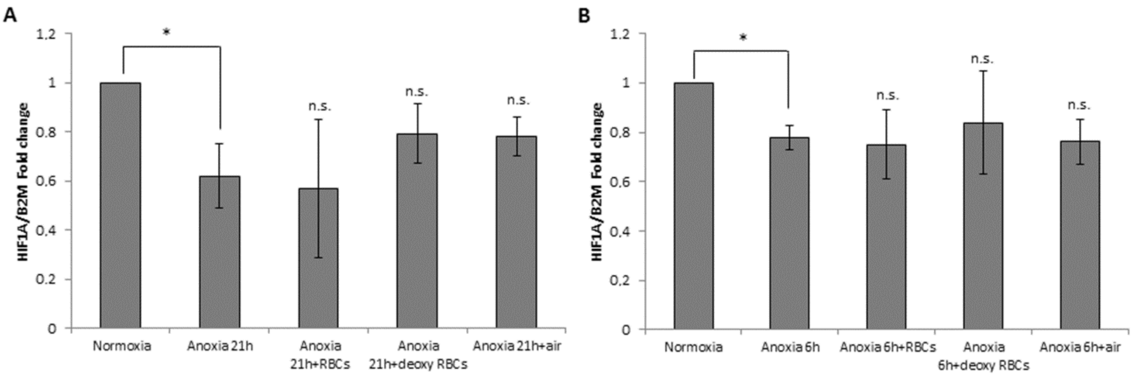

Supplementary Figure 1. HIF1A mRNA levels in cell extracts of MM6 cells (A) and human macrophages (B) after the incubation with RBCs (10% Ht) deoxygenated or not. Values are expressed as mean±SD; n=3; *p<0.05 when compared with normoxic samples.
